# Ectopic ossification presenting as osteoid metaplasia in a salivary mucocele in a Shih Tzu dog

**DOI:** 10.1186/1746-6148-8-13

**Published:** 2012-02-01

**Authors:** Thaís R Fernandes, Fabrizio Grandi, Lidianne N Monteiro, Breno S Salgado, Rafael M Rocha, Noeme S Rocha

**Affiliations:** 1 Serviço de Patologia Veterinária, Departamento de Clínica Veterinária, Faculdade de Medicina Veterinária e Zootecnia, Universidade Estadual Paulista - UNESP, Botucatu, Brazil; 2 Departmento de Patologia, Faculdade de Medicina de Botucatu - Universidade Estadual Paulista - UNESP, Botucatu, Brazil; 3 Curso de Medicina Veterinária, Fundação de Ensino e Pesquisa de Itajubá,- FEPI, Itajubá, Brazil; 4 Departamento de Anatomia Patológica, Hospital A.C. Camar go, Fundação Antônio Prudente, São Paulo, Brazil

## Abstract

**Background:**

Salivary mucocele is an accumulation of saliva in a single or multiloculated cavity lined by connective tissue that is contiguous to a salivary gland-duct complex and is the most common condition affecting the salivary glands in dogs. Occasionally, different types of metaplastic lesions, such as squamous and osseous metaplasia - which are rare lesions in animals - can be observed in association with salivary mucocele.

**Case presentation:**

A right facial enlargement was suddenly observed in a 4-year-old non-spayed female Shih-Tzu dog. The lesion presented itself as a soft and fluctuant mass located in the right side of the face near to the neck. Histologically, the mass consisted of a cavitary formation without an epithelial lining. Additionally, microscopic examination revealed the presence of osteoid-producing cells which gave rise to areas of bone formation, probably induced by irritation due to the presence sialoliths. Such cells and bone formations were also present in the cavity wall, consequently leading us to classify the condition as a salivary mucocele with osseous metaplasia.

**Conclusions:**

In the present case, the pathogenesis was probably associated with the presence of sialoliths, which can behave as etiological agents for the metaplastic lesion. The occurrence of osteoid metaplasia is a rare peculiar condition in the canine salivar y gland, and due to the rarity and lack of information about this specific disease, no clinical data can yet be associated with the development of salivary mucocele with osseous metaplasia in dogs.

## Background

Salivary gland disorders are uncommon diseases of dogs, with an overall reported incidence of less than 0.3 percent [1]. Salivary mucocele in an accumulation of saliva in a single or multiloculated cavity lined by connective tissue [2]. This cavity is contiguous to a salivary gland-duct complex [3] and is the most common condition affecting the salivar y glands in dogs [4]. Salivary mucoceles can reveal differentiation in several types of tissues, such as those represented by squamous [5] and osseous metaplasia [6,7]. There are only two reported cases of osteoid metaplastic salivary mucocele in the dog [6,7], none of them in the Shih Tzu breed. Accordingly, the aim of this article is to describe a case of this rare variation of salivary mucocele in a female Shih Tzu dog.

## Case description

**Figure 1:**
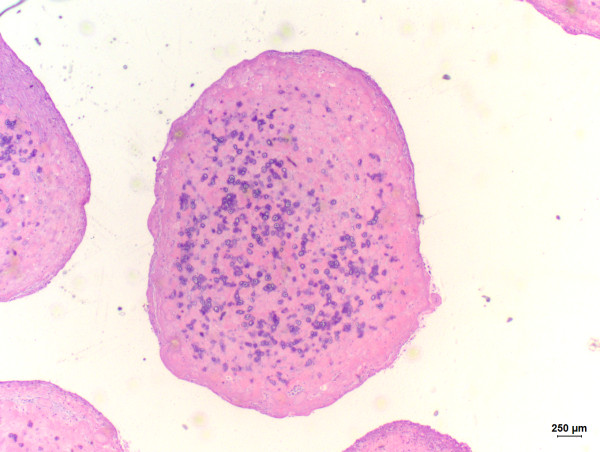
**Sialolith observed within the mucocele**. Hematoxylin and eosin (bar = 250 μm).

**Figure 2:**
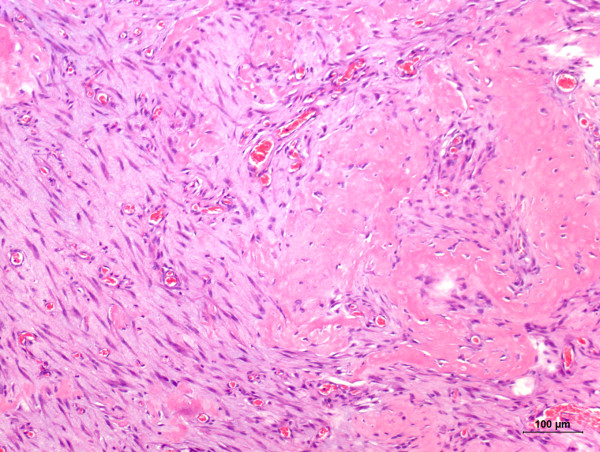
**Connective tissue composed by neoformed blood vessels, macrophages, lymphocytes, and active fibroblasts surrounded by an accentuated eosinophilic stroma characterizing a recent desmoplasia**. Hematoxylin and eosin (bar = 100 μm).

**Figure 3:**
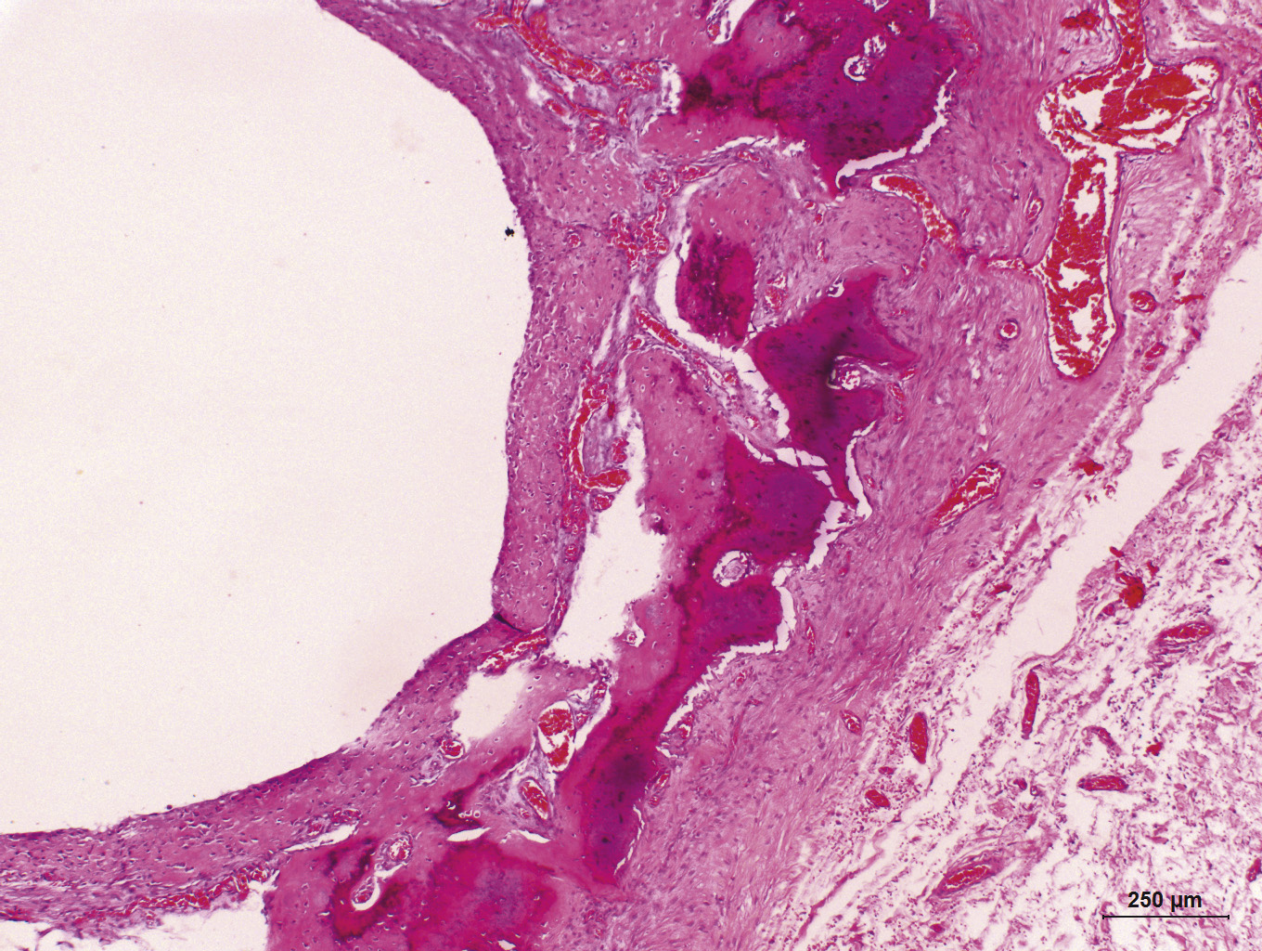
**Osteoid-producing cells giving rise to areas of bone formation**. Hematoxylin and eosin (bar = 250 μm).

A 4- year-old, non-spayed female Shih-Tzu dog was admitted to the School of Veterinar y Medicine and Animal Science from the São Paulo State University - UNESP, Botucatu, Brazil, for a soft and fluctuant non-ulcerated right facial swelling measuring 10 × 10 × 2 cm. The lesion was located in the neck near to the mandibular gland. Hematological and serum biochemistry findings revealed no alterations in blood urea nitrogen, creatinine and total protein concentrations. However, hyperalbuminemia and hypoglobulinemia were detected. An excisional biopsy with salivary gland resection was performed and the excised mass was submitted to the Veterinary Pathology Service of the same institution for histopathological examination. Grossly, the excised mass was cystic and thin-walled. Additionally, the presence of sialoliths was detected on gross examination. Fragments were formalin-fixed, routinely processed, and paraffin- embedded. Subsequently, 4 μm thick sections were obtained and stained with hematoxylin and eosin for histopathological examination. Histologically, the mass consisted of a cavitary formation without epithelial lining, leading us to diagnose the lesion as a salivary mucocele. Additionally, sialoliths located inside the cyst were observed on microscopic examination (Figure 1). Connective tissue composed by blood vessels, macrophages, lymphocytes, and active fibroblasts was also detected in the salivary mucocele, characterizing an association of the primary lesion with granulation tissue. The cells observed in such tissue were surrounded by an eosinophilic stroma, thus characterizing desmoplasia (Figure 2). Osteoid-producing cells which were giving rise to areas of bone formation were also present in the cyst wall (Figure 3), consequently leading us to suggest occurrence of ectopic ossification in association with the sialocele. The initial outcome of the resection treatment was satisfying, since one-year after the surgery the dog did not reveal any other signs of disease.

## Discussion

Salivary mucoceles are uncommon salivary gland disorders in dogs that do not have breed or sex predilection [1]. However, this condition is reported to be usually observed in French Poodles, German shepherd dogs, Dachshunds, and Australian Silky Terriers [3,8]. Salivary mucoceles can be classified as subcutaneous, sublingual, pharyngeal, or periorbital salivary mucoceles according the anatomic location [4] and affected salivary gland/duct. They must be differentiated from sialoadenitis, sialoadenosis, salivary gland neoplasms, sialoliths, abscesses, foreign bodies, hematomas, enlarged lymph nodes, and congenital cysts since they are the major diseases that affect the salivary gland of the dog [5,9,10]. The only way to adequately differentiate between those processes is through histological analysis, consequently highlighting histopathology as an indispensable method for reaching the correct diagnosis [5]. Microscopically, salivary mucoceles can occasionally reveal the presence of metaplastic lesions such as osteoid metaplasia; however, this condition seems to be a rare lesion in dogs and other species of veterinary interest because few cases have been reported to date [6,7]. Despite this, it is still possible to correctly suggest the occurrence of a salivary mucocele based on the presenting clinical signs (fluctuant, thin-walled lesion in the sublingual, intermandibular/cervical, or pharyngeal region) and other clinical findings such as aspiration of saliva-like fluid that may be transparent, but more often has a brownish color. Metaplastic lesions are presumptive reversible changes in which one adult cell type is replaced by another adult cell type. This lesion may also represent an adaptive substitution of cells more sensitive to stress by cell types that are better able to withstand an adverse environment. In cases of osteoid metaplasia, the condition is suggested to occur in fibrous connective tissue cells that undergo osteogenic differentiation and give rise to bone where it is normally not present [11]. This process is a subtype of ectopic ossification, which has two other subtypes: heterotopic ossification and osseous choristoma. Osseous choristoma is a lesion that is microscopically characterized by normal bone in abnormal sites [11]. On the other hand, heterotopic ossification is an alteration that usually occurs as secondary to metastatic mineralization from systemic disease or dystrophic mineralization [11]. Ectopic ossification can occur virtually anywhere [11-17] and, although being rare in salivary glands, has been reported in dogs with salivary mucocele presenting as OM [6,7]. In the present case, only mesenchymal cells undergoing osteogenic cell differentiation could be observed in the wall of the salivary mucocele and evidence neither from metastatic nor dystrophic mineralization were detected, similarly to what was found by other authors [6,7]. In this case, salivary sialoliths were grossly and microscopically observed. Such sialoliths can develop as a result of deposition of mineral salts around a nidus of bacteria, mucus, or desquamated cells [2]. Salivary stagnation, increased alkalinity of the saliva, increased calcium content of the saliva, infection or inflammation of the salivary duct or gland, and physical trauma to the salivary duct or gland may predispose to sialolith formation [18,19]. The pathogenesis of the process observed in the present case was not clearly understood; however, trauma and chronic inflammation could be considered as possible causes, since sialoliths and foreign bodies have been considered to be etiological agents in the development of salivary mucoceles in dogs [20,21]. There are only two reported cases of osteoid metaplasia salivary mucoceles in dogs to date [6,7], none of them in the Shih Tzu dog. This case report highlights a rare peculiarity within a salivary mucocele in a dog but due to the rarity and lack of information about this specific disease, no clinical data can yet be associated with the development of salivary mucocele with osseous metaplasia in dogs.

## Conclusions

In the present case, the pathogenesis was probably associated with the presence of sialoliths, which can behave as etiological agents for the metaplastic lesion due to trauma and inflammation. Histological analysis allowed us to classify the process as occurrence of granulation tissue associated with an osteoid metaplastic lesion in a salivary mucocele, consequently characterizing an ectopic ossification in this case.

## Consent

Written informed consent was obtained from the patient's owner for publication of this case report and any accompanying images. A copy of the written consent is available for review by the Editor-in-Chief of this journal.

## Competing interests

The authors declare that they have no competing interests.

## Authors' contributions

TRF performed the analysis and interpretation of cytologic and histologic findings, photographed images and helped to write the manuscript. FG, LNM, BSS participated in clinical research, helped to write the manuscript and has contributed to the analysis and interpretation of cytology and histology and for the preparation of the manuscript. RMR conducted clinical research and was responsible for collecting samples. NSR is the supervisor responsible for the case report and the review of the manuscript. All authors read and approved the final manuscript.
